# Correction: Dai et al. The *tae-miR164*-*TaNAC6A* Module from Winter Wheat Could Enhance Cold Tolerance in Transgenic *Arabidopsis thaliana*. *Plants* 2025, *14*, 2849

**DOI:** 10.3390/plants15010041

**Published:** 2025-12-23

**Authors:** Ziyao Dai, Xiaoyan Yang, Wenwang Shan, Yiou Hao, Da Zhang, Kankan Peng, Qinghua Xu

**Affiliations:** College of Life Science, Northeast Agricultural University, Harbin 150030, China; daiziyao224776@163.com (Z.D.); yang20230223@163.com (X.Y.); shiq18279@gmail.com (W.S.); 17804620855@163.com (Y.H.); zhangda@neau.edu.cn (D.Z.); pengkankan@neau.edu.cn (K.P.)

In the original publication [1], there was an error in Figure 7 as published. The photo, which was from the Recovery of STTM-*tae-miR164*-1, was incorrect. The corrected Figure 7 appears below.

We state that the scientific conclusions are unaffected. This correction was approved by the Academic Editor. The original publication has also been updated.

## Figures and Tables

**Figure 7:**
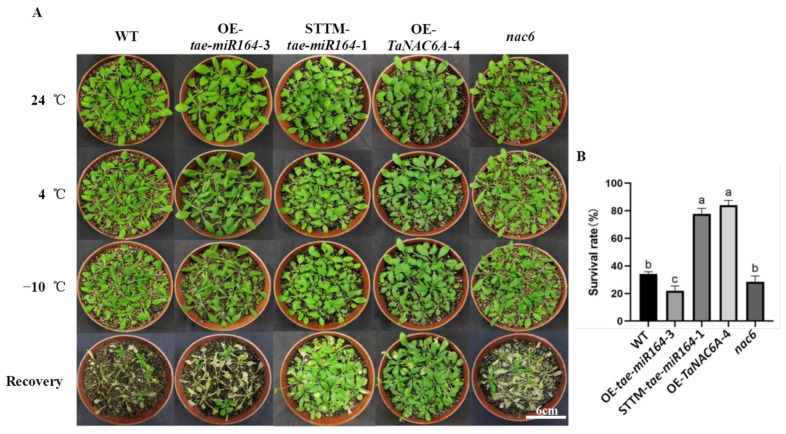
Phenotypes and survival rates of Arabidopsis plants under freezing stress. (**A**) Plant phenotypes; scale bars = 6 cm; (**B**) survival rates. Values represent the means ± SEs (*n* = 30). Different lowercase letters indicate significant differences between treatments (*p* < 0.05), determined by two-way ANOVA.
