# Supplementary material for: The tae-miR164-TaNAC6A Module from Winter Wheat Could Enhance Cold Tolerance in Transgenic Arabidopsis thaliana
Source: Plants (Basel). 2025 Sep 12;14(18):2849. doi: 10.3390/plants14182849 (PMC12473215; doi:10.3390/plants14182849)
Supplement: Supplementary file 1 [file plants-14-02849-s001.zip › plants-3819203-supplementary.pdf]

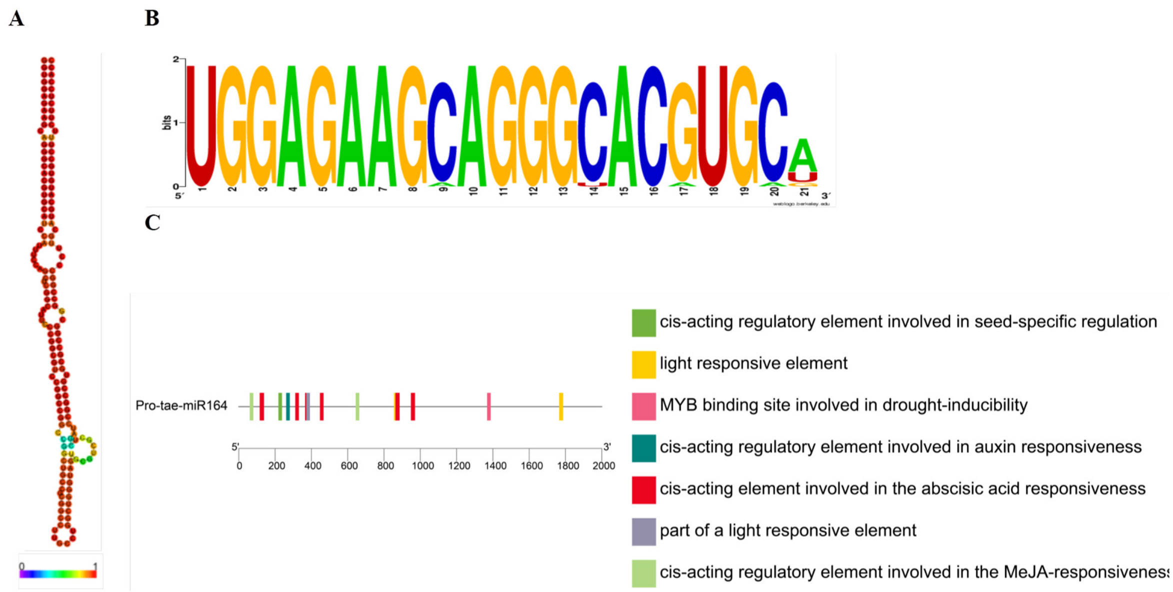

Figure S1. Bioinformatics analysis of *tae-miR164* and its promoters. (A) Secondary structure prediction; (B) Base conservation analysis of *tae-miR164* maturation sequence. (C) Promoter cis-element analysis.

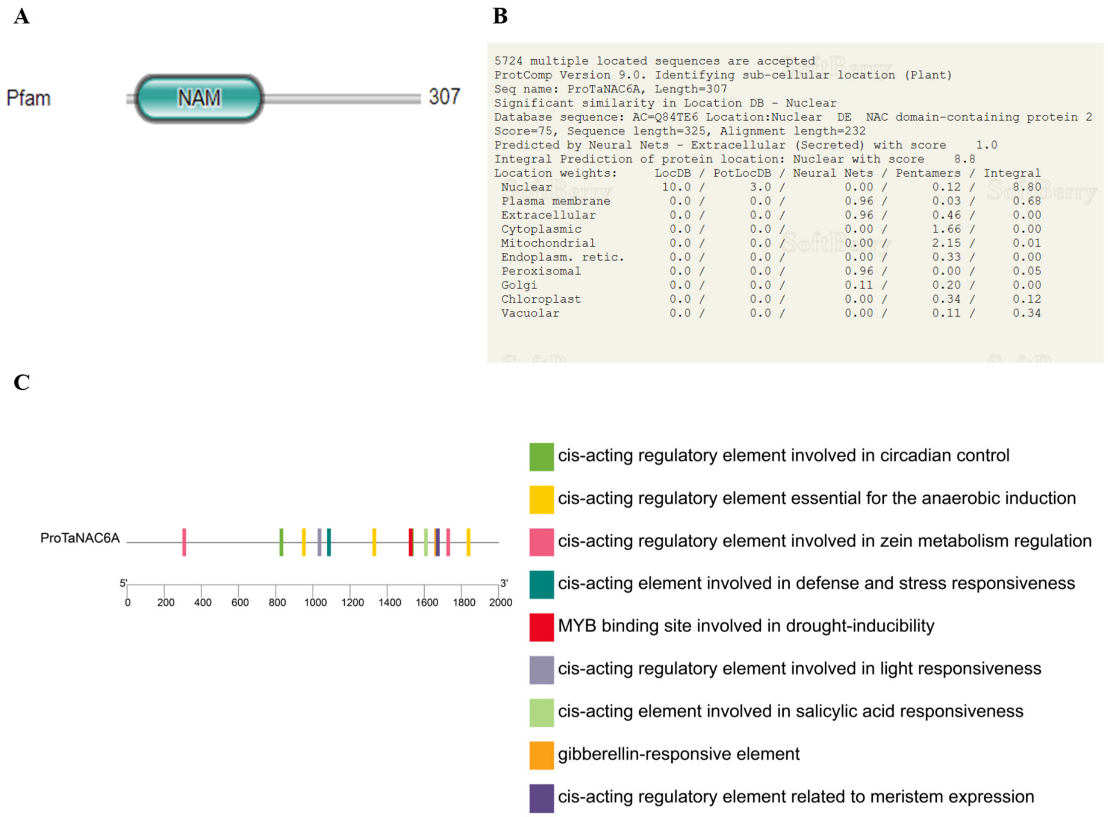

Figure S2. Bioinformatic analysis of the TaNAC6A structure and cis-element in its promoter. (A)Protein conserved domain analysis; (B) Subcellular localization prediction; (C) Promoter cis-element analysis.

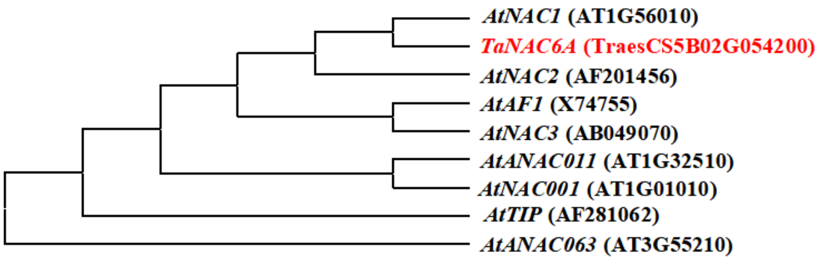

Figure S3. Phylogenetic tree conducted by MEGA11 software. Using the neighbor-joining method, with the Bootstrap value set to 1000. TaNAC6A (TraesCS5B02G054200) colored in red and AtNAC1 (AT1G56010) are positioned on the same branch.

Table S1. sequences of primers for qRT-PCR of wheat and Arabidopsis plants

	Primer	Sequence
wheat	*TaActin* F *(housekeeping gene)*	5′-CCTTAGTACCTTCCAACAGATGT-3′
	TaActin R *(housekeeping gene)*	5′-CCAGACAACTCGCAACTTAGA-3′
	Q *tae-miR164* F	5′-TGGAGAAGCAGGGCACGTGCA-3′
	Q *TaNAC6A* F	5′-CAAGTGCCCTGCTTCTTCC-3′
	Q *TaNAC6A* R	5′-TGCCATTACTGTTGCCTGT-3′
Arabidopsis plants	*AtActin*F *(housekeeping gene)*	5′-TGTGCCAATCTACGAGGGTTT-3′
	AtActin R *(housekeeping gene)*	5′-TTTCCCGCTCTGCTGTTGT-3′
	AtCAT1 F	5′-AGGAGCCAATCACAGCC-3′
	AtCAT1 R	5′-TCAAGACCAAGCGACCA-3′
	AtCAT2 F	5′-CTTCAGCTGCCAGTCAATGC-3′
	AtCAT2 R	5′-GCAGGCGGAGTTGGATACTT-3′
	AtCAT3 F	5′-CCACTTGATGTGACCAAGATCTG-3′
	AtCAT3 R	5′-GTAGATTCCAGGAACCACAAGACC-3′
	AtSOD1 F	5′-AGGAAACATCACTGTTGGAGAT-3′
	AtSOD1 R	5′-GAGTTTGGTCCAGTAAGAGGAA-3′
	AtSOD2 F	5′-TCGTATCACTGGTCTCACTC-3′
	AtPER3 F	5′-GGAAATGTAAAAGATTTGGGAGCTTG-3′
	AtPER3 R	5′-TATTGACGAAGTCTGTAACAAAATCC-3′
	AtABI3 F	5′-TCCATTAGACAGCAGTCAAGGTTT-3′
	AtABI3 R	5′-GGTGTCAAAGAACTCGTTGCTATC-3′
	AtABI4 F	5′-GGGCAGGAACAAGGAGGAAGTG-3′
	AtABI4 R	5′-ACGGCGGTGGATGAGTTATTGAT-3′
	AtABI5 F	5′-CAATAAGAGAGGGATAGCGAACGAG-3′
	AtABI5 R	5′-CGTCCATTGCTGTCTCCTCCA-3′
	AtDREB1 F	5′-GCCGATCAGCCTGTCTCAAT-3′
	AtDREB1 R	5′-TCTGCCATATTAGCCAACAAACTC-3′
	AtDREB2 F	5′-AAGGGTCGAAGAAGGGGTTGT-3′
	AtDREB2 R	5′-CGAGCCAAAGGACCATACAT-3′

Table S2. Sequences of primers for promoter amplifying

Primer	Sequence
PromiR164 F	5’-ACGCATCTGGGAAAGTTGGA-3’
PromiR164 R	5’-GCAGAGCTGACACCACAACA-3’
ProTaNAC6A F	5’-GAGAGATGTCGATGAGCTTCC-3’
ProTaNAC6A R	5’-GAAGTGGTTCCAAGTAGAGCT-3’

Table S3. Sequences of primers for 5′RACE

3′Primer	Sequence
TaNAC6A OR (first cycle)	5′GAGCTCTACTTGGAACCACTTCTA-3′
TaNAC6A IR (second cycle)	5′-GTGACAAGAAGGTCCTTAAGGCAG-3′

* Primers only specificity to *TaNAC6A*.

Table S4. Sequences of primers for dual luciferase assay

Primer	Sequence
pre-tae-miR164 F	5′-CAGTGGTCTCACACC-3′
pre-tae-miR164 R	5′-CAGTGGTCTCAAGCG-3′
TaNAC6A F	5′-CAGTGGTCTCAGATC-3′
TaNAC6A R	5′-CAGTGGTCTCAAATT-3′

Table S5. List homologous genes of Arabidopsis for *TaNAC6A* gene

Query	Target	Description	Species	Name	Qcovs	Length	Identity	Evalue
TaNAC6A (TraesCS5B02G054200)	AT1G56010	NAC domain containing protein 1	Arabidopsis	NAC1	99.0	325	47.692	1.33e-90
	AT3G12977	AT3G12977	Arabidopsis	null	90.0	283	47.35	6.58e-80
	AT3G18400	NAC domain containing protein 58	Arabidopsis	NAC058	59.0	182	56.593	3.86e-68

* This analytical process conducted by WheatOmics (http://202.194.139.32/). Qcovs : Query Coverage Per Subject. Length : Alignment length. Identity : Percentage of identical matches. Positive : Percentage of positive-scoring matches. Evalue : Expect value.
